# Cobalt and manganese carboxylates for metal oxide thin film deposition by applying the atmospheric pressure combustion chemical vapour deposition process†

**DOI:** 10.1039/c8ra02288g

**Published:** 2018-04-25

**Authors:** B. S. M. Kretzschmar, K. Assim, A. Preuß, A. Heft, M. Korb, M. Pügner, T. Lampke, B. Grünler, H. Lang

**Affiliations:** Innovent e.V. Prüssingstraße 27B 07745 Jena Germany bg@innovent-jena.de +49-3641-2825-30 +49-3641-2825-28; Technische Universität Chemnitz, Faculty of Natural Sciences, Institute of Chemistry, Inorganic Chemistry 09107 Chemnitz Germany heinrich.lang@chemie.tu-chemnitz.de +49-371-531-21219 +49-371-531-21210; Technische Universität Chemnitz, Faculty of Mechanical Engineering, Institute of Material Sciences and Engineering, Materials and Surface Engineering 09107 Chemnitz Germany

## Abstract

Coordination complexes [M(O_2_CCH_2_OC_2_H_4_OMe)_2_] (M = Co, 4; M = Mn, 5) are accessible by the anion exchange reaction between the corresponding metal acetates [M(OAc)_2_(H_2_O)_4_] (M = Co, 1; M = Mn, 2) and the carboxylic acid HO_2_CCH_2_OC_2_H_4_OMe (3). IR spectroscopy confirms the chelating or μ-bridging binding mode of the carboxylato ligands to M(ii). The molecular structure of 5 in the solid state confirms a distorted octahedral arrangement at Mn(ii), setup by the two carboxylato ligands including their α-ether oxygen atoms, resulting in an overall two-dimensional coordination network. The thermal decomposition behavior of 4 and 5 was studied by TG-MS, revealing that decarboxylation occurs initially giving [M(CH_2_OC_2_H_4_OMe)_2_], which further decomposes by M–C, C–O and C–C bond cleavages. Complexes 4 and 5 were used as CCVD (combustion chemical vapour deposition) precursors for the deposition of Co_3_O_4_, crystalline Mn_3_O_4_ and amorphous Mn_2_O_3_ thin films on silicon and glass substrates. The deposition experiments were carried out using three different precursor solutions (0.4, 0.6 and 0.8 M) at 400 °C. Depending on the precursor concentration, particulated layers were obtained as evidenced by SEM. The layer thicknesses range from 32 to 170 nm. The rms roughness of the respective films was determined by AFM, displaying that the higher the precursor concentration, the rougher the Co_3_O_4_ surface is (17.4–43.8 nm), while the manganese oxide films are almost similar (6.2–9.8 nm).

## Abstract 2

Complexes [M(O_2_CCH_2_OC_2_H_4_OMe)_2_] (M = Co, Mn) were synthesized characterised regarding their thermal behaviour. They were used as precursors for deposition of rough Co_3_O_4_ and smooth Mn_2_O_3_/Mn_3_O_4_ thin films *via* the CCVD process.

## Graphical abstract

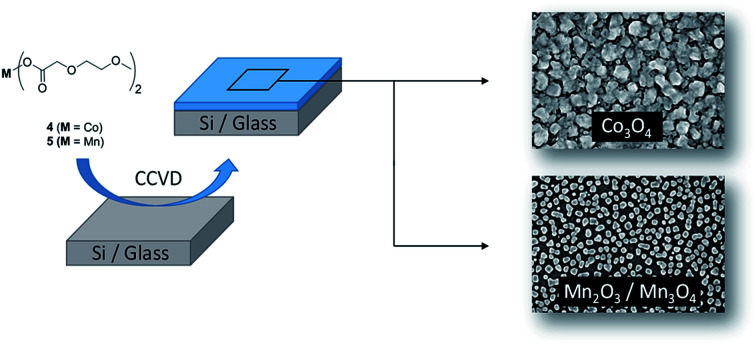

## 1 Introduction

The modification of solid surfaces by using thin film deposition techniques is one of the main research areas in modern nanotechnology, which is considered as an interdisciplinary research field involving physics, chemistry and material sciences.^1^ Recently, the preparation of transition metal oxide thin films from manganese and cobalt has gained great interest, due to their wide application range.^2^ Manganese oxide thin films, for instance, can be used as antibacterial coatings,^1,3^ as electrochemical materials,^4^ as NTC material in sensors,^5^ or as electrode material in batteries and super-capacitors.^6,7^ Similarly, cobalt oxide is of importance, for example, as a blue pigment in ceramics,^8^ as an anode material in lithium-ion batteries,^9^ and as electrode material in super-capacitors.^10^ Most of these applications are owing to the capability of the elements, manganese and cobalt, to occupy different oxidation states. Thus, they are enabled to form different transition metal oxides including M_2_O_3_, M_3_O_4_ (M = Mn or Co) and MnO_2_, which allows their use in diverse areas of application.

During the last few years, various deposition methodologies such as physical vapour deposition (PVD),^11,12^ chemical vapour deposition (CVD)^13,14^ and atomic layer deposition (ALD)^10^ have been established to fabricate manganese oxide and cobalt oxide thin films. Within these methods solid and liquid precursors, like cobalt(ii) nitrate hexahydrate^9^ and dicarbonylcyclopentadienylcobalt^10^ for Co_3_O_4_, or manganese(ii) chloride tetrahydrate^4^ and manganese sulphate^15^ for Mn_3_O_4_ layer formation were established. These deposition techniques are vacuum-based and hence are cost intensive. In addition, the precursors must meet certain requirements. For instance, CVD and ALD precursors have to exhibit high vapour pressures in order to ensure their transformation into the gas phase without being thermally decomposed. Furthermore, straightforward surface chemistry and low decomposition temperatures are needed.

In contrast, the non-vacuum-based combustion chemical vapour deposition (CCVD) process is a very cost effective deposition technique and hence makes it a lucrative method for metal and/or metal oxide deposition.^16^ By changing the CCVD parameters, the film morphology can be easily controlled in surface roughness, surface area and porosity. Accordingly, ultrathin SiO_*x*_ layers on glass substrates were deposited under varied conditions (substrate temperature, burner passes and substrate velocity) creating a barrier against glass leaching.^16^ Similarly prepared silicon dioxide layers provide an alternative to conventional surface treatment for reactive metals, Schinkinger *et al.* investigated CCVD nanoscaled SiO_2_ films on zinc-coated steel as an interface between an organic coating and the metal substrate.^17^ Moreover, thin ZnO layers have been deposited by CCVD using non-volatile zinc nitrate as metal oxide source.^18,19^ WO_*x*_ thin films are a further example, which are utilized in “smart windows”, due to their capability of changing their colour from colourless to blue in an electrochromic cell.^19^ Dhonge *et al.* investigated the morphology alteration of Al_2_O_3_ thin films by varying the substrate temperature within the CCVD process.^20^

Not only metal oxides can be deposited *via* the CCVD process, but also noble metal layers as this was realized for the deposition of silver on glass.^21,22^

We herein present the straightforward synthesis of [M(O_2_CCH_2_OC_2_H_4_OMe)_2_] (M = Co, Mn) and their successful application as CCVD precursors for the deposition of cobalt and manganese oxide layers on silicon (100) wafers and 4 mm soda lime silicate float glass (air face) substrates. The chemical and morphological structure of the as-deposited metal oxide thin films is discussed depending on the deposition conditions applied.

## 2 Materials and methods

### 2.1 Instruments and material

The carboxylic acid 3 as well as cobalt(ii) acetate tetrahydrate (1) and manganese(ii) acetate tetrahydrate (2) were purchased from Sigma-Aldrich and were used without further purification.

IR spectroscopy was carried out with a FT Nicolet IR 200 instrument. For high-resolution mass-spectrometric studies a microOTOF QII Bruker Daltonik workstation utilizing the Apollo II electrospray ionization (ESI) source was applied. Elemental analyses of the complexes were conducted with a Thermo FlashAE 1112 analyzer. The melting points were determined by a Gallenkamp MFB 595 010 M melting point device. TG, DSC and TG-MS coupled studies were carried out with a Mettler Toledo TGA/DSC1 1600 system with a MX1 balance coupled with a Pfeifer Vacuum MS Thermostar GSD 301 T2 mass-spectrometer. PXRD measurements of the respective TG residues were performed with a STOE STADI-P diffractometer equipped with a germanium (111) monochromator and Cu K_α_ radiation (*λ* = 1.5406 Å, 40 kV, 40 mA).

Single crystal X-ray diffraction analysis data of 5 were collected with an Oxford Gemini S diffractometer using graphite-monochromated Mo K_α_ radiation (*λ* = 0.71073 Å) at 110.8(2) K. The molecular structure was solved by direct methods utilizing SHELXS-13 and full-matrix least squares procedures on *F*^2^.^23–25^ All non-hydrogen atoms were refined anisotropically and a riding model was employed in the refinement of the hydrogen atom positions. Graphics of the respective structure were created by using ORTEP and SHELXTL.^26^ CCDC-no. 1573318 contains the supplementary crystallographic data for this work.†

The combustion chemical vapor deposition experiments were carried out by a home-built CCVD system. The precursor feed rate of the peristaltic pump can be varied between 0.1 mL min^−1^ and 12 mL min^−1^. The propane air ratio is controlled by a gas supply unit (50 MD-1B) from Sura Instruments and can be switched between 1 : 16 and 1 : 24. The temperature of the propane–air flame ranges from 1400 to 2300 °C. Velocity of the sample holder of up to 600 mm s^−2^ could be realized. The working distance can be varied from 10–100 mm and a maximum substrate temperature of 600 °C is possible.

The crystal structures of the as-deposited thin films were identified by the Grazing Incidence X-ray diffraction (GIXRD) technique with a D8 Discover diffractometer (Bruker) using monochromized Co K_α_ radiation (*λ* = 1.79026 Å). The angle of incidence was 0.5°. Oxidation states were estimated by using X-ray photoelectron spectroscopy (XPS) (Thermo Fischer ESCALAB 250 Xi) with a monochromized Al K_α_ radiation wavelength of 8.34 Å. The film morphology was studied by using Scanning Electron Microscopy (SEM) (Supra 60-32-10 Zeiss) with an InLens detector and Atomic Force Microscopy (AFM) (Ultra Objectiv, SIS today Bruker) in a non-contact mode.

### 2.2 Synthesis of cobalt(ii)- and manganese(ii) carboxylates 4 and 5

The title complexes were synthesized accordingly to a preparation procedure published previously.^27^ In a typical synthesis experiment, two equiv. of the carboxylic acid 3 were added to a suspension of the respective metal(ii) acetate 1 or 2 in 50 mL of toluene. The reaction mixture was then heated to 60 °C and the azeotropic mixture of water, toluene and acetic acid was removed by an azeotropic distillation under reduced pressure (130 mbar). After all volatiles were removed in vacuum, the crude products were washed thrice with diethyl ether (each 30 mL). Complex 4 was obtained as purple^28^ and 5 as pink solid.

#### Cobalt(ii) 2-(2-methoxyethoxy)acetate (4)

Yield: 99% based on the respective metal(ii) acetate. The analytical data obtained are in agreement with those ones published previously.^28^ IR data (KBr): *ν* = 2878 (w), 1752 (w), 1610 (s), 1419 (s), 1325 (w), 1199 (w), 1103 (s), 1036 (w), 945 (w), 705 (m). Mp: 114 °C.

#### Manganese(ii) 2-(2-methoxyethoxy)acetate (5)

Yield: 99% based on the respective metal(ii) acetate. Mp: 147 °C. Anal. calcd. for C_10_H_18_O_8_Mn (321.18): C 37.40, H 5.65; found C 37.61, H 5.72. IR data (KBr): *ν* = 2877 (m), 1754 (w), 1588 (s), 1416 (s), 1325 (m), 1296 (w), 1246 (w), 1199 (w), 1088 (s), 1032 (m), 943 (m), 896 (w), 850 (w), 711 (m). HR-MS (ESI-TOF): calcd. for C_10_H_18_O_8_Mn *m*/*z* = 322.0460 [M + H]^+^; found *m*/*z* = 322.0454.

#### Crystal data of 5

C_10_H_18_MnO_8_, *M* = 321.18 g mol^−1^, crystal dimensions 0.30 × 0.30 × 0.20 mm, monoclinic, *P*2_1_/*c*, *λ* = 0.71073 Å, *a* = 8.7974(5) Å, *b* = 6.6960(4) Å, *c* = 23.1544(15) Å, *β* = 93.145(5)°, *V* = 1361.91(14) Å^3^, *Z* = 4, *ρ*_calcd_ = 1.566 mg m^−3^, *μ* = 1.000 mm^−1^, *T* = 110.8(2) K, *θ* range 2.989–24.995°, 5188 reflections collected, 2369 independent reflections (*R*_int_ = 0.0285), *R*_1_ = 0.0614, *wR*_2_ = 0.1519 (*I* > 2*σ*(*I*)).

### 2.3 Preparation of cobalt oxide and manganese oxide thin films by the CCVD process

**Fig. 1:**
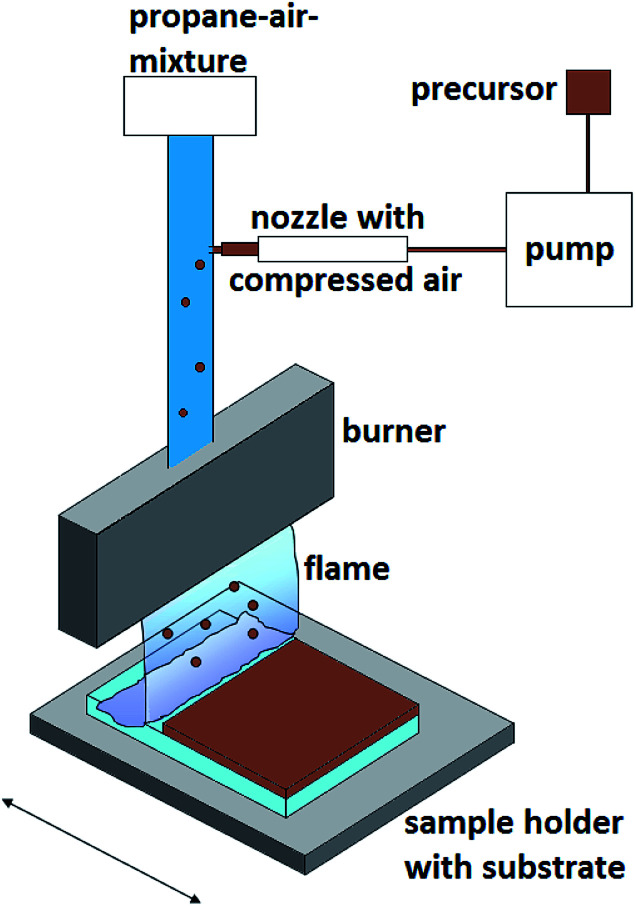
Schematic CCVD setup.

The burner was fed with a gaseous fuel–air (3.6 mL min^−1^ propane and 70 mL min^−1^ air) mixture doped with the synthesized precursors. The liquid precursor (for more details see below) was sprayed directly into the fuel–air-mixture as aerosol droplets by using a compressed air nozzle (Fig. 1).

### 2.4 Deposition

A burner of 10 cm width was used in the CCVD deposition studies. The sample holder was moved 30 times through the flame of the burner. After ten burner passes there was a holding time of about 10 s. As substrates silicon (100) wafers and 4 mm soda lime silicate float glass (air face), previously cleaned with isopropyl alcohol and dried with oil-free compressed air, were used. A peristaltic pump with a precursor feed rate of 1.5 mL min^−1^ was used for the general deposition procedure. Constant process parameters: propane air ratio of 1 : 19.4 for a stoichiometric combustion, velocity of the table 50 mm s^−2^, and working distance 20 mm. A substrate temperature of 400 °C and a post-annealing time of 2 h were applied (Table 1). Samples were prepared by varying the precursor concentration (0.4, 0.6 and 0.8 M) in methanol. After cooling to ambient temperature, the coated substrates were cleaned in an isopropyl alcohol ultrasonic bath for 10 min. The samples were then rinsed with distilled water and dried with oil-free compressed air.

**Table 1:** Deposition parameters for the formation of cobalt oxide and manganese oxide layers Co_3_O_4_ and Mn_2_O_3_/Mn_3_O_4_ using complexes 4 and 5 as CCVD precursors

Parameter	
Flow rate propane [L min^−1^]	3.6
Flow rate air [L min^−1^]	70
Flow rate precursor [mL min^−1^]	1.5
Precursor concentration [mol L^−1^]	0.4, 0.6, 0.8
Distance substrate/burner [mm]	20
Velocity of the substrate holder [mm s^−1^]	50
Temperature of the substrate holder [°C]	400
Number of passes	30
Holding time after 10 burner passes [s]	10
Post-annealing time [h]	2

## 3 Results and discussion

### 3.1 Synthesis and characterization of [M(O_2_CCH_2_OCH_2_CH_2_OMe)_2_] (4, 5)

**Scheme 1:**
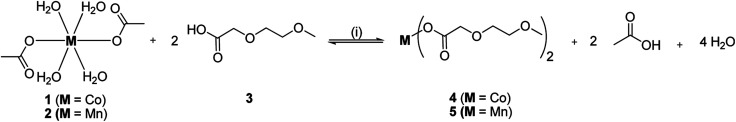
Synthesis of 4^28^ and 5 by the reaction of 1 and 2 with 3 ((i) toluene, 60 °C, 5 h, azeotropic distillation).

The ethylene glycol-functionalized cobalt(ii) and manganese(ii) carboxylates 4 and 5 were synthesized by the anion exchange reaction of the respective metal acetates 1 and 2 with 2-(2-methoxyethoxy)acetic acid (3) (Scheme 1, experimental). After appropriate work-up, complexes 4 and 5 were obtained in virtually quantitative yields. In comparison to 1 and 2, complexes 4 and 5 show significant higher solubility in polar solvents such as water, methanol, ethanol and dichloromethane. Complex 5 is hydrophilic and converts within hours to a viscous oil, if exposed to air humidity.

Since the characterization of 4 was in detail described previously in ref. 28, only 5 will be discussed in the following.

The identity of the as-prepared paramagnetic manganese(ii) carboxylate complex 5 was confirmed by IR spectroscopy, elemental analysis and high-resolution ESI-TOF mass spectrometry (experimental). Moreover, its thermal behaviour was investigated by thermogravimetric-coupled mass-spectrometry (TG-MS). The molecular structure in the solid state is reported.

**Fig. 2:**
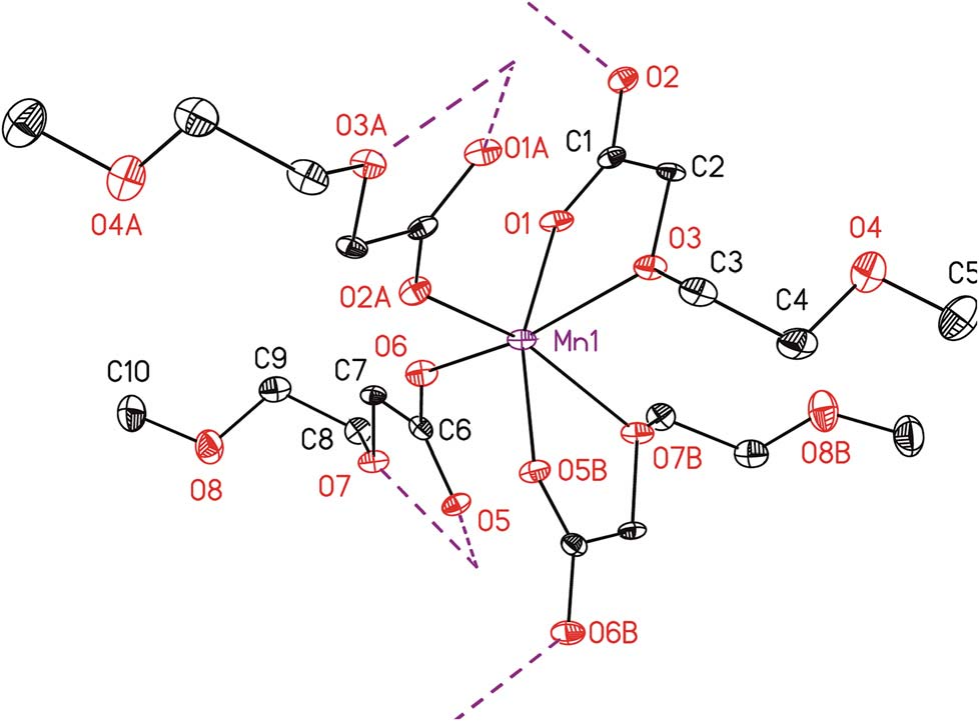
ORTEP (50% probability level) showing a part the molecular structure of 5 with the atom numbering scheme. Hydrogen atoms have been omitted for clarity. Symmetry operation for generating equivalent atoms: (A) −*x* + 1, *y* + 1/2, −*z* + 1/2; (B) −*x*, *y* + 1/2, −*z* + 1/2.

IR spectroscopy was applied to investigate the coordination mode of the carboxylato ligands to the manganese ion in 5. It is quantified that the C–O stretching frequencies directly correlate with a certain type of binding.^29^ There exist three different binding motifs that can be distinguished by the interpretation of the respective IR data: uni-dentate, chelating and bridging-bidentate coordination. In fact, the determining factor is the separation (Δ*ν*_CO_2__) between the asymmetric and symmetric frequencies (Δ*ν*_CO_2__ = *ν*_asym_ − *ν*_sym_). Therefore, an ionic reference system consisting of the potassium or sodium salt of the same or a related carboxylic acid is required. The bridging and chelating ligands possess a similar or smaller Δ*ν*_CO_2__ value, while the uni-dentate arrangement exhibits mostly a significantly increased Δ*ν*_CO_2__ value, when compared to the respective reference sample.^29^ To determine the binding motif of the carboxylic ligand in 5 and compare it with 4, the potassium salt of 2-[2-(2-methoxyethoxy)ethoxy]acetic acid was used as reference system, as the same compound was also applied for cobalt carboxylate 4 in previous works.^28^ The respective Δ*ν*_CO_2__ value was found to be 185 cm^−1^. Relative to this value the separation of 172 cm^−1^ for 5 is considered indicative for a bridging or chelating fashion. This was additionally confirmed by the molecular structure determination of 5 (Fig. 2), confirming a μ-bridging coordination type of the carboxylato ligand.

High resolution ESI-TOF-mass spectrometric studies evidenced the formation of the complex, since the corresponding protonated molecular ion [M + H^+^] with *m*/*z* = 322.0454 (cald. *m*/*z* = 322.0460) could be detected.

The melting point of 5 was measured to 147 °C showing that it possesses a higher melting temperature than 4, which melts at 114 °C.^28^

The molecular structure of 5 in the solid state was determined by single crystal X-ray analysis. Suitable crystals were obtained from a concentrated toluene solution containing 5 at ambient temperature. The respective ORTEP is depicted in Fig. 2. Relevant crystal and structural refinement data are shown in the experimental section. Selected bond lengths (Å) and angles (°) are summarized in Table 2.

**Table 2:** Selected bond lengths [Å] and angles [°] of 5

Bond lengths/Å		Angles/°	
Mn–O1	2.139(3)	O1–Mn–O5	161.67(13)
Mn–O2	2.123(3)	O2–Mn–O7	157.62(13)
Mn–O3	2.237(3)	O3–Mn–O6	159.99(13)
Mn–O5	2.153(3)	O1–Mn–O3	72.96(13)
Mn–O6	2.126(3)	O2–Mn–O6	94.29(13)
Mn–O7	2.314(3)	O3–Mn–O7	82.43(13)
C1–C2	1.515(7)	O5–Mn–O7	70.90(13)
C3–C4	1.501(8)	O5–Mn–O6	104.32(13)
		O1–Mn–O6	87.61(13)

Complex 5 crystallizes in the monoclinic space group *P*2_1_/*c*. One manganese atom and two carboxylato ligands constitute the asymmetric unit, whereas three different oxygen atoms (O1, O3 and O6) of these two ligands coordinate to the Mn(ii) ion. The octahedral coordination sphere at manganese, containing four ligands (Fig. 2), was generated by two symmetry operations (A) −*x* + 1, *y* + 1/2, −*z* + 1/2 and (B) −*x*, *y* + 1/2, −*z* + 1/2. The respective octahedron is distorted, which is clarified by the smallest *trans* angle of O2–Mn–O7 with 157.62(13)°, whereas no distinction between the axial and equatorial position can be made. Moreover, *cis* angles are shortened as well, as, for example, the chelate angles O5–Mn–O7 and O1–Mn–O3 are only 70.90(13) and 72.96(13)°, respectively. The manganese ion is coordinated by carboxylic (O1, O2, O5 and O6) and α-ether oxygen atoms of the ethylene glycol chain (O3 and O7). The ether and the *cis*-positioned carboxylic oxygen atoms are chelate-bonded to Mn forming a five-membered cycle. This coordination behavior is in agreement with the structure of complexes bearing similar ligands, *e.g.* [Co(O_2_CCH_2_OMe)_2_(H_2_O)_2_].^30^ In contrast, the β-ether oxygen atoms O4 and O8 are of non-coordinating character, which is most probably due to their spatial distance and the saturated coordination sphere of manganese. The Mn–O bond distances for the α-ether oxygen atoms Mn1–O3 and Mn1–O7 are with 2.237(3) and 2.314(3) Å significantly longer than those for the carboxylate oxygen atoms Mn1–O1, Mn1–O2, Mn1–O5 and Mn1–O6 (Table 1).

Due to the μ^2^-bridging motif of the carboxylato ligands, as also confirmed by IR studies (*vide infra*), a two-dimensional polymer structure is formed, whereas the layer growth is parallel to the A–B plane. The ethylene glycol chains direct up- and downwards in the vacant space between these A–B planes, hence they are located between the polymeric layers. No interaction was found between the layers and the ethylene glycol chains. The minimum distance between manganese atoms of adjacent layers is 11.165 Å (Fig. SI1†).

### 3.2 Thermal behaviour

**Fig. 3:**
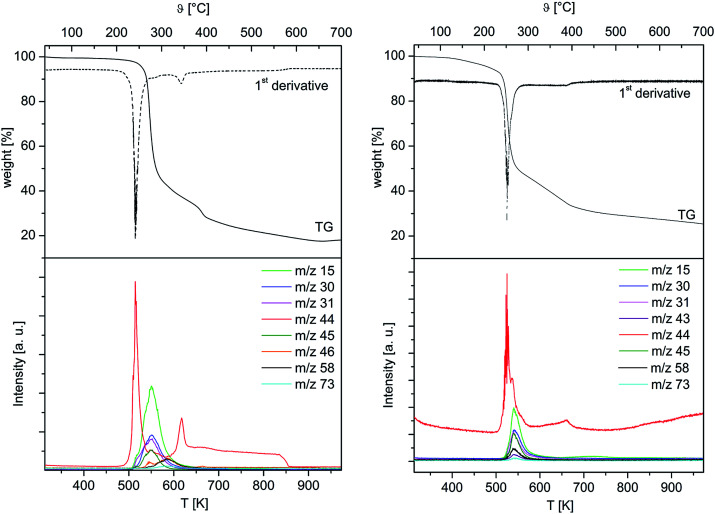
Thermogravimetry (TG) (top) and selected mass spectrometric scans (bottom) of 4 (left) and 5 (right). *m*/*z* = 15 (CH_3_^+^), 30 (C_2_H_6_^+^), 31 (CH_3_O^+^), 43 (C_2_H_3_O^+^), 44 (CO_2_^+^), 45 (C_2_H_5_O^+^), 46 (C_2_H_6_O^+^), 58 (C_3_H_6_O^+^), 73 (C_4_H_9_O^+^).

To obtain first information about the thermal behaviour and gain a deeper insight into the decomposition mechanism of 4 and 5, TG-MS studies on these complexes were carried out. The studies were conducted under an atmosphere of argon using a gas flow of 60 mL min^−1^. A heating rate of 5 °C min^−1^ was chosen for 4, while 2.5 °C min^−1^ was used for 5 in order to avoid violent gas evolution during the heating process. The respective TG-MS traces including the weight loss, their first derivate and the ion current curves of the appropriate mass-to-charge ratios (*m*/*z*) of both complexes are depicted in Fig. 3. As it can be seen from the TG plots, the initial decomposition step gives rise to the typical fragment *m*/*z* = 44 for 4 and 5, confirming the decarboxylation as initial decomposition step. Subsequently, it is assumed that the intermediates [M(CH_2_OC_2_H_4_OMe)_2_] (M = Co, Mn) are generated *in situ*. By increasing the temperature >250 °C, the thermal degradation of these species occurs, which results from the formation of fragments such as *m*/*z* = 15 (CH_3_^+^), 31 (OCH_3_^+^), 45 (C_2_H_5_O^+^), 58 (C_3_H_6_O^+^) and 73 (C_4_H_9_O^+^). Since the release of these ions is time-delayed, it is suggested that the thermal degradation of the respective intermediates [M(CH_2_OC_2_H_4_OMe)_2_] occurs, when the initial decarboxylation process is completed.

Considering the first derivatives of the corresponding TG traces, it was found that for 4 and 5 an additional decomposition step takes place in the temperature range of 600 and 700 °C. This leads to somewhat increase of the CO_2_^+^ concentration in this temperature interval, which is indicative for the combustion of the carbon residues.

The appropriate TG-MS residues have been investigated by PXRD studies. As it can be seen from Fig. SI2 (ESI†), it was found that the residue obtained from thermolysis of 4 consists of crystalline CoO [00-048-1719], which was confirmed by three reflections at 36.49, 42.39 and 61.49° indicating the {111}, {200} and {220} crystal orientations, respectively. The residue from 5, however, comprises of two different manganese oxide phases, as corresponding reflections for MnO [00-007-0230] and Mn_3_O_4_ [00-018-0803] were found (Fig. SI3, ESI†). While only two reflections at 40.54 and 58.72° were identified referring to MnO, the presence of the Mn_3_O_4_ phase was verified by reflections such as, 28.96, 32.41, 36.04 and 60.02° pointing to the {112}, {103}, {211} and {224} crystal orientations.

Since the thermal decomposition of both complexes is not completed under the measurement conditions applied (800 °C) the CCVD technique is a quit suitable process to complete the thermal degradation processes. The high temperature of the flame can lead to the comprehensive combustion of the precursor resulting in the deposition of thin films on the substrates used.

### 3.3 Film characterization

The as-deposited layers obtained by the CCVD technique using 4 and 5 as precursor complexes on glass substrates are pale brown and transparent. By increasing the precursor solution concentration from 0.4 *via* 0.6 to 0.8 M the colour intensity increases, whereas the transparency decreases. Gracing incidence X-ray diffraction (GIXRD) measurements were performed to investigate the crystal structure of the deposited manganese oxide and cobalt oxide thin films.

**Fig. 4:**
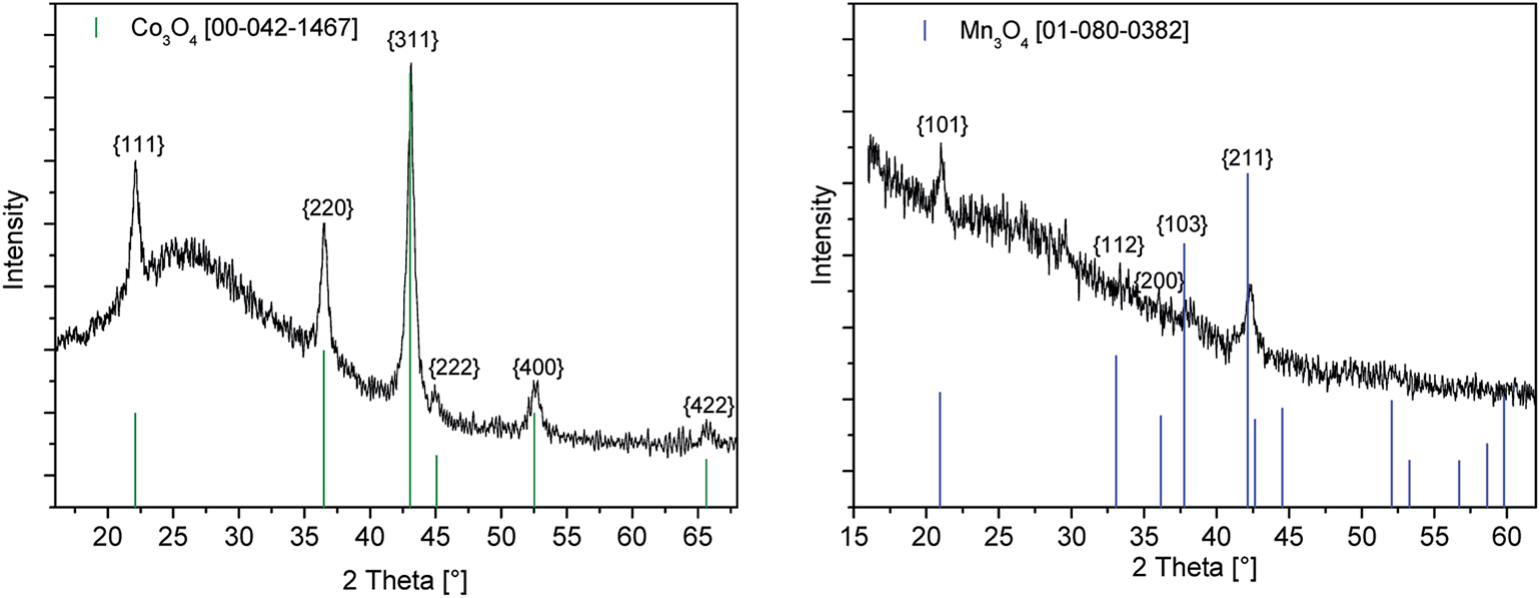
GIXRD spectra of the as-deposited cobalt oxide (left) and manganese oxide (right) layers by the CCVD process using 4 and 5 as precursors (precursor concentration 0.8 M).

The cobalt oxide layer obtained from the 0.8 M precursor solution shows the diffraction according to the cubic lattice of Co_3_O_4_ [00-042-1467] (Fig. 4). The peaks found at 2*θ* = 22.11, 36.53, 43.13, 44.99, 52.56 and 65.66° represent the respective {111}, {220}, {222}, {311}, {400} and {422} planes of the crystal structure and are in good agreement with literature values.^31^

The GIXRD diagram of the deposited manganese oxide thin film using a precursor concentration of 0.8 M is depicted in Fig. 4 (right). The three diffraction peaks observed at 2*θ* = 21.00, 38.18 and 42.38° are indicative for the {101}, {211} and {202} crystal orientations of the hausmannite phase Mn_3_O_4_ [01-080-0382].

**Fig. 5:**
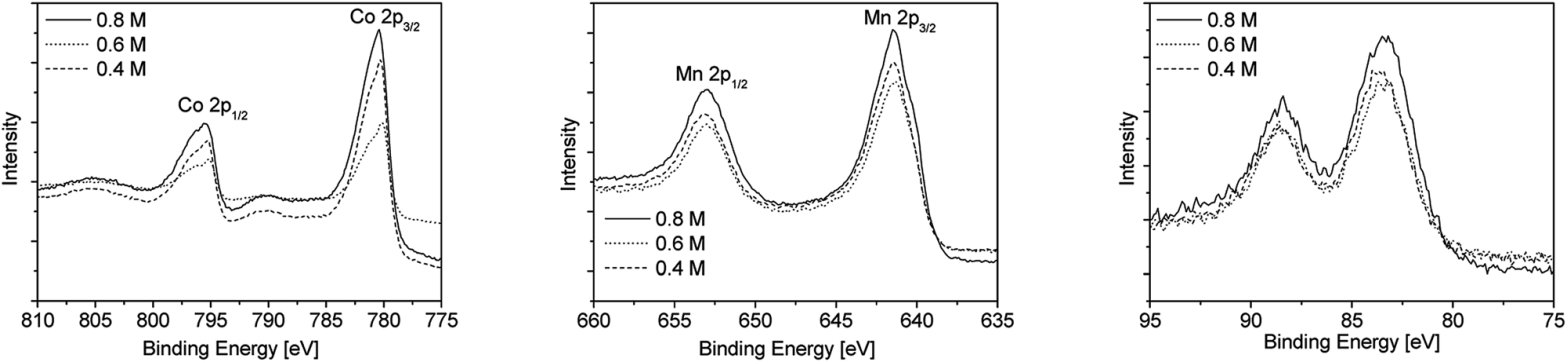
Co 2p (left), Mn 2p (middle) and Mn 3s (right) XPS spectra of the cobalt oxide and manganese oxide layers deposited on float glass as substrate using different precursor concentrations.

In addition XPS studies were performed to gain more information on the chemical composition of the as-obtained metal oxide films. The XPS spectra of the cobalt oxide and manganese oxide layers deposited on float glass as substrate using different precursor concentrations are depicted in Fig. 5. The XPS survey spectra of cobalt oxide and manganese oxide films using the 0.8 M precursor solutions are exemplary shown in Fig. SI4 and SI5 (ESI†). For the cobalt oxide layers satellite features were observed due to the Co^2+^ and Co^3+^ oxidation states in Co_3_O_4_. The XPS spectra show two main peaks located at 780.3 and 795.4 eV, which correlates with Co 2p_1/2_ and Co 2p_3/2_, respectively^32^ (Fig. 5, left). Using the Auger parameter (1552.9 eV), the thin layers were identified as Co_3_O_4_.^33^

It was found that the binding energies for Mn 2p_1/2_ and Mn 2p_3/2_ at 641.5 and 653 eV are in agreement with the Mn_3_O_4_ phase.^34^ In contrast to that the XPS detail spectra (Fig. 5, middle) show no satellites between the Mn 2p_1/2_ and Mn 2p_3/2_ peaks at 647 eV, which is quite surprising since the satellite appearance corresponds to divalent manganese.^35^ However, by determination of the Auger parameter (1226.8 eV), the presence of manganese oxide Mn_2_O_3_ is suggested.^36^ The Mn 3s peak (Fig. 5, right) splits into two multiplet-split components (*ca.* 5.5 eV), emphasising the presence of a trivalent Mn state (Mn_2_O_3_).^37^

According to the GIXRD and XPS measurements it is assumed that the layer contains most probably crystalline Mn_3_O_4_ and amorphous Mn_2_O_3_.

**Fig. 6:**
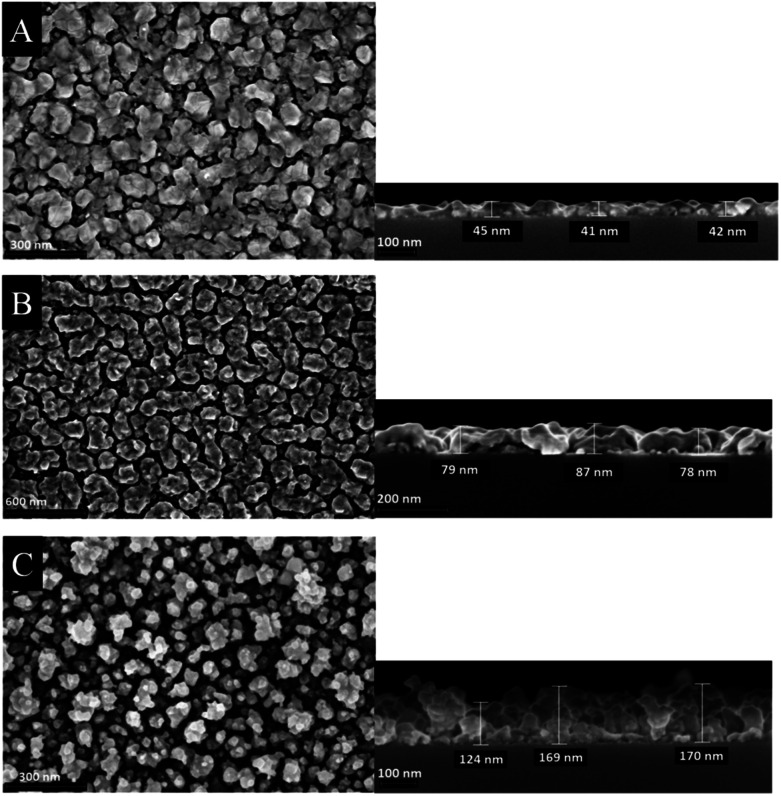
Top view (left) and cross section (right) SEM images of cobalt oxide thin films deposited on silicon wafers using different precursor concentration (A) 0.4 M, (B) 0.6 M and (C) 0.8 M.

**Fig. 7:**
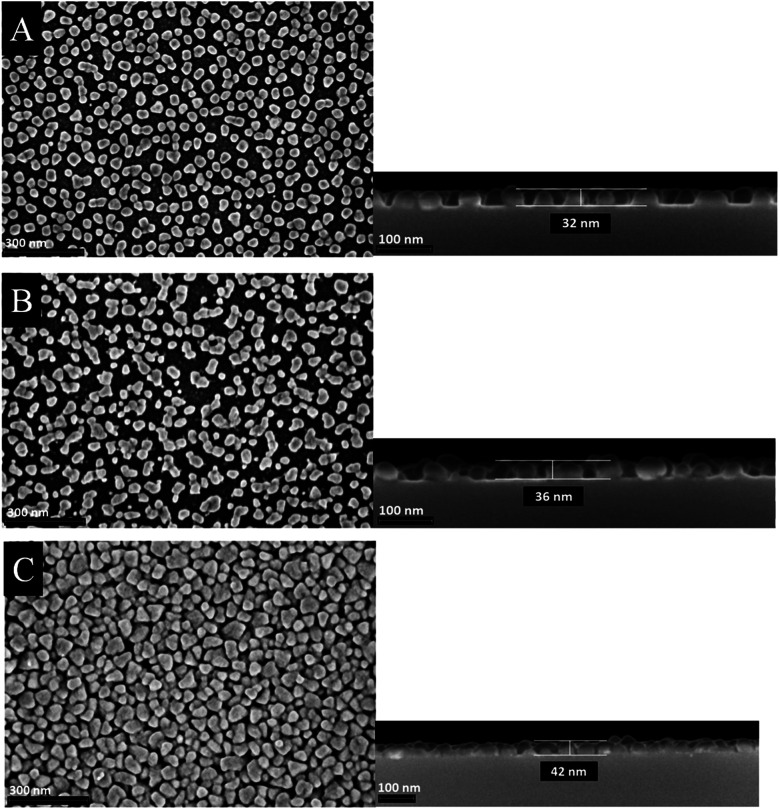
Top view (left) and cross section (right) SEM images of manganese oxide thin films deposited on silicon wafers using different precursor concentration (A) 0.4 M, (B) 0.6 M and (C) 0.8 M.

The morphology of the as-deposited layers were examined by Scanning Electron Microscopy (SEM). Additionally, cross-section studies have been carried out in order to determine the film thicknesses. Fig. 6 and 7 show the top view as well as the cross section SEM images of all thin metal oxide layers obtained from the different precursor concentrations (see earlier, 0.4–0.8 M).

The film thickness of the as-deposited cobalt oxide layers increases from 42 up to 170 nm by increasing the precursor concentration from 0.4 to 0.8 M (Fig. 6). The higher porosity is evidenced by the SEM top view studies. It is suggested that during the CCVD process initially a monolayer is formed, whereas further deposited particles agglomerate on its surface. This refers to a combination of the Franck-van-der-Merwe and Volmer–Weber growth mechanisms, which is also known as Stranski–Krastanov growth.^38^ Vaz *et al.*, for example, studied the growth stages of epitaxial Co_3_O_4_ films grown on α-Al_2_O_3_ single crystals. The low energy electron diffraction (LEED) pattern symmetry remains to the substrate till 17 Å. With higher layer thickness (34 Å) it starts resembling of Co_3_O_4_. These results suggest that the Co_3_O_4_ layers were generated following the Stranski–Krastanov growth mechanism.^39^

In contrast to the cobalt oxide films, the manganese oxide thin films appear smoother (Fig. 7). The film thicknesses slightly increase from 32 to 42 nm by increasing the precursor concentration. From SEM images it can be seen that small isolated islands are formed rather than closed thin films. Therefore, it is suggested that the film growth follows a different mechanism. The CCVD process of the formation of manganese oxide layers obeys most probably the Volmer–Weber growth mechanism, due to the fact that the crystallization and growth of seeds is probably much faster than the formation of a monolayer. Similar observations were made earlier, as, for example, for the deposition of manganese oxide films by the atomic layer deposition process.^10^

**Fig. 8:**
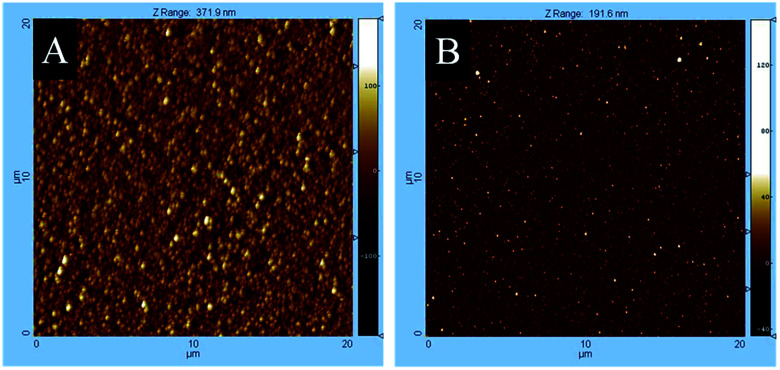
AFM mappings of (A) cobalt oxide and (B) manganese oxide thin films deposited by CCVD using a 0.8 M precursor concentration of 4 and 5, respectively.

The AFM measurements maintain the morphological results investigated by SEM. Fig. 8 shows the surface morphology of cobalt oxide and manganese oxide films obtained from the precursor concentration of 0.8 M. The determined rms roughnesses of all samples are presented in Table 3. As it can be seen from this table, the surface roughness differs significantly. While the cobalt oxide samples exhibit a rms roughness between 17.4–43.8 nm, the manganese containing layers only reveal values from 6.2 to 9.8 nm. As shown in Table 3, there is no simple correlation between film morphology and the nature of the substrate for the cobalt oxide films found. The manganese containing layers have a higher roughness on glass than on a silicon substrate.

**Table 3:** Root mean square roughness of cobalt oxide and manganese oxide thin films deposited on silicon wafers and float glass using 4 and 5 as CCVD precursors

rms roughness [nm]		Concentration [M]		
		0.8	0.6	0.4
Co_3_O_4_	Float glass	43.8 ± 1.2	30.4 ± 0.9	17.4 ± 0.5
	Si wafer	41.4 ± 1.1	30.4 ± 1.2	20.5 ± 0.4
Mn_*x*_O_*y*_	Float glass	9.8 ± 0.2	8.5 ± 0.2	8.2 ± 0.4
	Si wafer	8.3 ± 0.1	6.9 ± 0.3	6.2 ± 0.2

## 4 Conclusion

Metal carboxylates [M(CO_2_CH_2_OC_2_H_4_OMe)_2_] (M = Co, 4; M = Mn, 5) were prepared by a straightforward anion exchange reaction by reacting [M(OAc)_2_(H_2_O)_4_] (M = Co, 1; M = Mn, 2) with the carboxylic acid HCO_2_CH_2_OC_2_H_4_OMe (3). Beneficial is that these complexes show a higher solubility as compared to the cobalt or manganese starting materials and hence are predestined as CCVD precursors for metal oxide deposition. From IR spectroscopy it can be concluded that the carboxylato ligands in 4 and 5 are chelate or bridge-bonded to M. The bridging coordination mode of the carboxylic groups in 5 was confirmed by single crystal X-ray investigation. Thermogravimetric and thermogravimetric-coupled mass spectrometric studies have been carried out on 4 and 5 showing that the thermal decomposition of both complexes is initialized by decarboxylation followed by M–C, C–C and C–O cleavages. The PXRD studies of the TG residues of 4 confirmed the formation of crystalline CoO [00-048-1719], whereas the obtained residue from 5 consisted of the two different manganese oxides MnO [00-007-0230] and Mn_3_O_4_ [00-018-0803].

Complexes 4 and 5 were successfully used as precursors for the deposition of cobalt oxide and manganese oxide thin films by applying the cost-effective Combustion Chemical Vapor Deposition process (CCVD). This technique allows the easy control of the layer thickness as well as the film porosity. The obtained films were characterized by GIXRD, XPS, SEM and AFM studies. In terms of the respective cobalt oxide films, GIXRD and XPS evidenced the formation of Co_3_O_4_. In contrast, two different phases were found in the as-deposited manganese oxide layers. While only reflections belonging to the Mn_3_O_4_ hausmannite phase were detected by GIXRD, the respective XPS measurements confirm the presence of Mn_2_O_3_, too. Therefore, we suppose that the manganese oxide layers are comprised of crystalline Mn_3_O_4_ and amorphous Mn_2_O_3_.

Morphological investigations of the metal oxide thin films were carried out by SEM and AFM. The thicknesses of the cobalt oxide layers range from 42 to 170 nm. Concurrently, an increase of surface roughness could be obtained by increasing the precursor concentration from 0.4 to 0.8 M. In contrary, the manganese oxide thin films are much smoother, whereby the respective layer thicknesses vary from 32 to 42 nm, while the porosity of the films remains constant by increasing the precursor concentration.

Due to the morphological and chemical state of the as-deposited films, a potential application field could be the use in electronic devices such as sensors or as electrode material for super-capacitors.

## Compliance with ethical standards

The authors thank Cornelia Kowol and Prof. Dr Stefan E. Schulz from the Fraunhofer Institute for Electronic Nano Systems (ENAS) for the SEM measurements. Manuel Monecke and Prof. Dietrich Zahn, Institute of Physics, TU Chemnitz are acknowledged for measuring the XPS spectra. To Lutz Mertens and Prof. Dr Michael Mehring we are grateful for carrying out the PXRD measurements. The publication costs of this article was funded by the German Research Foundation/DFG and the Technische Universität Chemnitz in the funding programme open access publishing.

## Conflicts of interest

The authors declare that they have no conflict of interest.

## Supplementary Material

RA-008-C8RA02288G-s001

RA-008-C8RA02288G-s002
