# Supplementary material for: Cobalt and manganese carboxylates for metal oxide thin film deposition by applying the atmospheric pressure combustion chemical vapour deposition process
Source: RSC Adv. 2018 Apr 25;8(28):15632–40. doi: 10.1039/c8ra02288g (PMC9080101; doi:10.1039/c8ra02288g)
Supplement: RA-008-C8RA02288G-s001 [file RA-008-C8RA02288G-s001.pdf]

Electronic Supplementary Material (ESI) for RSC Advances.
This journal is © The Royal Society of Chemistry 2018

Cobalt and Manganese Carboxylates for Metal Oxide Thin Film Deposition by Applying the Atmospheric Pressure Combustion Chemical Vapour Deposition Process

B. S. M. Kretzschmar[a], K. Assim[b], A. Preuß[b], A. Heft[a], M. Korb[b], M. Pügner[c], T. Lampke[c], B. Grünler[a*], H. Lang[b*]

a) Innovent e.V., Prüssingstraße 27B, 07745 Jena (Germany)

b) Technische Universität Chemnitz, Faculty of Natural Sciences, Institute of Chemistry, Inorganic Chemistry, 09107 Chemnitz (Germany)

c) Technische Universität Chemnitz, Faculty of Mechanical Engineering, Institute of Material Sciences and Engineering, Materials and Surface Engineering, 09107 Chemnitz (Germany)

**Corresponding authors:*

Email: bg@innovent-jena.de; phone: +49(0)3641-2825-28; fax.: +49(0)3641-2825-30

Email: heinrich.lang@chemie.tu-chemnitz.de; phone: +49(0)371-531-21210; fax.: +49(0)371-531-21219.

Content

Wire-firm model of complex **5** 2
PXRD patterns of the TG residues 3
XPS patterns of the deposited thin films 4

Wire-firm model of complex 5

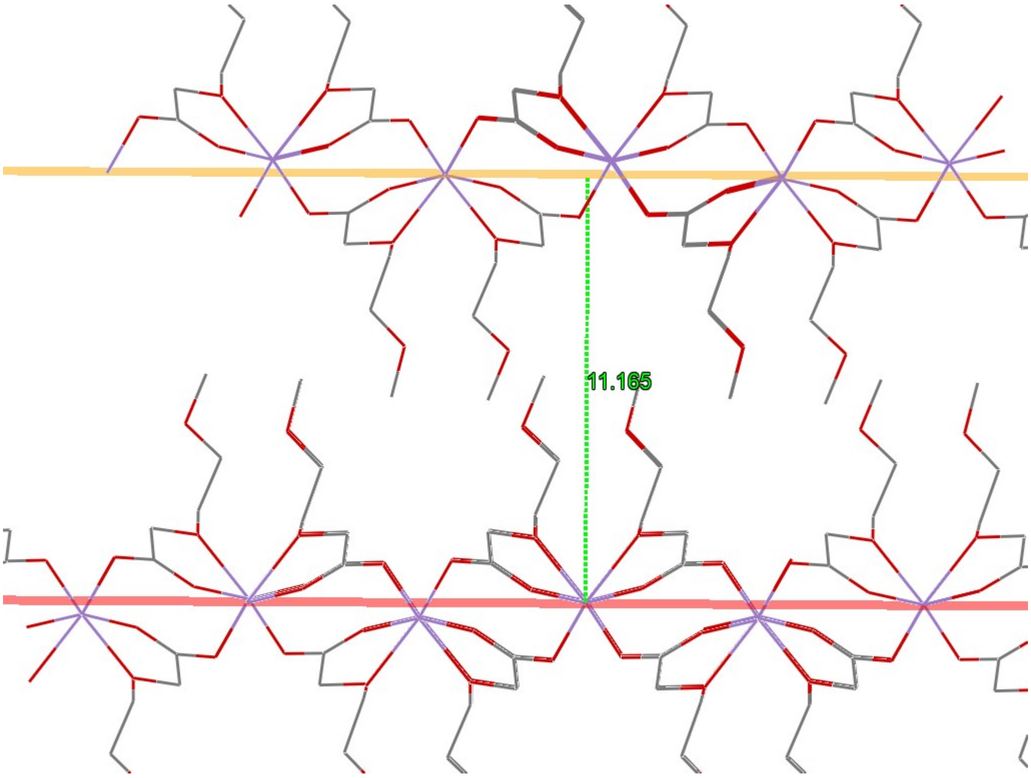

Figure SI1. Wire-firm model of complex **5** showing the distance of 11.165 nm between manganese atoms of adjacent layers.

PXRD patterns of the TG residues

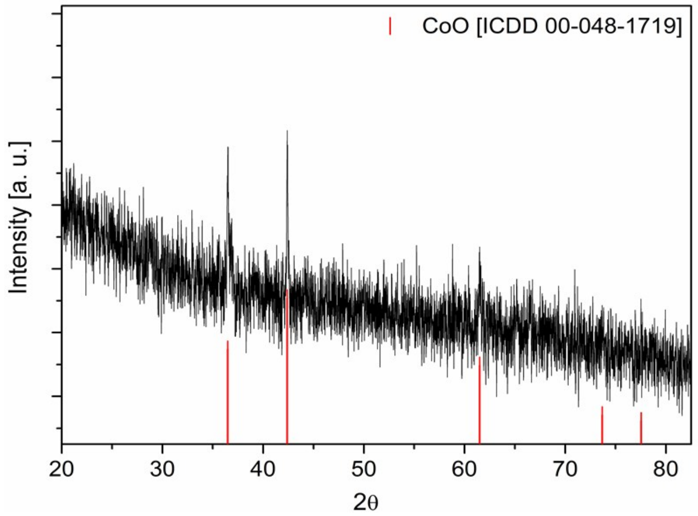

Figure SI2. PXRD of the residue obtained by the TG-MS study of **4** under argon (gas flow, 60 mL; heating rate, 5 K min^{-1}).

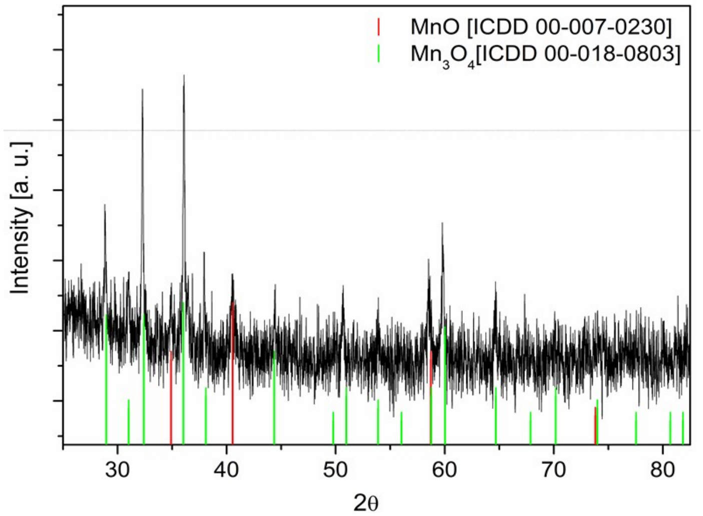

Figure SI3. PXRD of the residue obtained by the TG-MS study of **5** under argon (gas flow, 60 mL; heating rate, 2.5 K min^{-1}).

XPS patterns of the deposited thin films

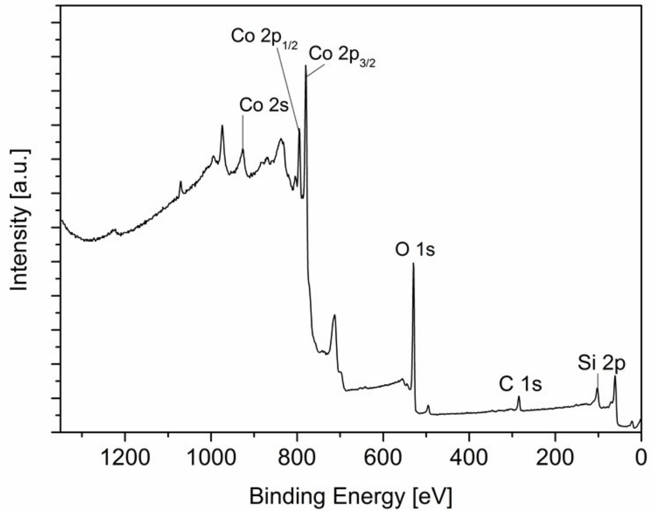

Figure SI4. XPS survey spectra of the cobalt oxide thin layer deposited on a glass substrate using a 0.8 M precursor concentration of **4**.

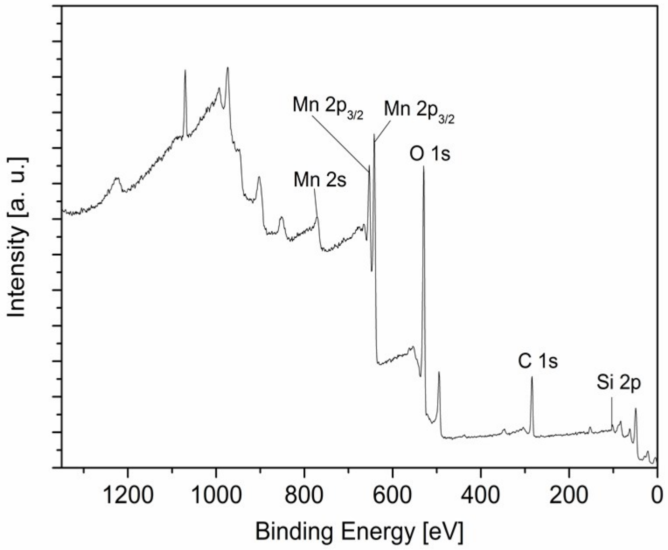

Figure SI5. XPS survey spectra of the manganese oxide thin layer deposited on a glass substrate using a 0.8 M precursor concentration of **5**.
